# Nutritional Assessment of Dietary *Bt* and *Cp4EPSPS* Proteins on the Serum Biochemical Changes of Rabbits at Different Developmental Stages

**DOI:** 10.3389/fnut.2018.00049

**Published:** 2018-06-05

**Authors:** Ibrahim B. Salisu, Ahmad A. Shahid, Qasim Ali, Abdul Q. Rao, Tayyab Husnain

**Affiliations:** ^1^Department of Animal Science, Faculty of Agriculture, Federal University Dutse, Dutse, Nigeria; ^2^Centre of Excellence in Molecular Biology, University of the Punjab, Lahore, Pakistan

**Keywords:** cottonseed meal, genetically modified cotton, nutritional assessment, rabbits, serum biochemistry

## Abstract

In recent years, the influence of genetically modified (GM) cotton expressing different types of *Bt* and *EPSPS* genes has been attested in term of reduced application of pesticides and insecticides coupled with improved cotton production. Although the cultivation of GM cotton has been authorized by the regulatory authorities of various countries in the world, based on the biosafety studies reported by most of the GM cotton producers, yet the information on its safe use are inadequate. In order to support the issues on food safety, it is therefore mandatory to conduct further safety assessment studies on GM cotton for each independent transgenic event on the basis of case assessment rule. In the present study, the effects of different doses of dietary GM cotton seed expressing *Bt* and *EPSPS* genes were studied on the level of serum biochemical in albino rabbits (*Oryctolagus cuniculus*). The rabbits were fed a diet containing different levels of GM cotton seeds (i.e., 20, 30, and 40% w/w) respectively mixed with standard diet for 180 days. During the course of the study, various serum enzymes, electrolytes, proteins, glucose and serum total cholesterol were examined at specific time intervals (0, 45, 90, 135, and 180) days. The results showed non-significant (*P* > 0.05) dose dependent effects in most of the evaluated serum biochemical parameters. Although, the results in some of the serum biochemistry were significantly different (*P* < 0.05) among the groups, however, they were not biologically significant, since all the differences were within the normal physiological range. These results thus, suggested that the GM cotton seed meal could be considered as safe as other conventional feed ingredients. The experimental evidence for the safe usage of GM cotton was highlighted in this study.

## Introduction

With the recent increase in world population coupled with income growth, the need for an improved food production became necessary. The global agricultural production need to be increased by 60% than that in the previous years by 2050 to combat the increasing food demand (1). The emergence of plant biotechnology, during the early 1980s, has currently been progressive intensely (2). Different plant varieties with improved characteristics such as resistance to insect, herbicides tolerance, and enhanced nutritional quality have been developed successfully. Recently there are more than 200 different transgenic crops having different characters approved for consumption in various countries (3). Genetically modified (GM) plants with a “*Bt*” gene expressing an insecticidal protein provide an improved technology for sustainable crop production. However, concern has been raised by the public over the food safety of these GM crops with respect to human consumption and production of animal products like meat, milk, and eggs from livestock raised on these GM crops (4). GM plant products are increasingly becoming part of human food-chain. In spite of this, feeding studies assessing the impact of GM-crops on human and animal health are comparatively less (5). The issue of utilization of feed ingredients derived from GM crops in animal rearing raises a considerable controversy. Numerous reports on this topic have been published in the past declaring no harmful effects on animal and human health (6), however, opposite opinions also exist and are experimentally documented (7–10). Research on food safety are also limited to some products and usually not on cotton. For instance, most of the earlier experiments were done on GM plants such as brinjal, corn, and soybean (11–14). Studies on GM cotton with respect to risk assessment are yet inadequate in proving its safety (15, 16). Therefore, it is the need of hour to generate comprehensive dietary risk assessment database for case-wise studies on GM cotton with various animals (wild, ruminant, and mono-gastric animals) (17).

Serum biochemical profile reflects nutritional, physiological, and pathological status of an individual (18–20). Biochemical parameters are inter-related with different body functions and alteration. Any impairment of serum biochemical indices can eventually result in structural and physiological abnormalities (21). Through the analyses of serum biochemical parameters, the function of vital organs like liver, kidney, heart, and pancreas can be determined easily. Serum biochemical examination, in general, allows for clinical investigation of various constituents of animal bodies. This is especially significant in rabbits, as they demonstrate complex clinical signs that often disguise the disease conditions (22).

Keeping in view the significance of biochemical components in animal, dietary ingredients are found to impart a considerable effect on serology (23). While studying and evaluating animal physiology and nutrition, laboratory tests can be applied to examine the model animals. The information regarding the effect of feeding dietary *Bt* and *Cp4epsps* proteins on the serum biochemical profile is scanty, particularly in case of rabbits. Therefore, this study was conducted to assess the dietary incorporation of transgenic cottonseed expressing *Bt* and *Cp4epsps* proteins on the serology of growing rabbits.

## Materials and methods

### Study area

Present studies were conducted at National Center of Excellence in Molecular Biology (CEMB), University of the Punjab, Lahore, Pakistan. The effect of triple genes cotton is studied on different biochemical parameters of the test animals.

### Test substance

The test substance is a mature seed of cotton (*Gossypium hirsutum)* variety (VH-289) genetically modified with binary insect resistant genes (*Cry 1Ac & Cry 2A*) and glyphosate herbicide tolerant gene (*EPSPS)*. The GM cotton seeds were provided by the repository at CEMB, University of Punjab, Lahore, Pakistan.

### Diet formulation

Diet for control and test animals were formulated according to the CEMB optimized procedure. The test substances (Cottonseeds) were crushed with the help of mechanical grinder following the removal of lint from the cotton boll. The seeds were crushed further to remove any remaining linters or strands of tiny cotton fibers. The seeds were then hulled and polished for releasing soft and highly edible protein. The hulled seeds were then mixed thoroughly with other types of feed ingredients using a mechanical mixer to make it suitable for the animals. Four different experimental diets were formulated to contain varying levels of cottonseed meal as a substitute for wheat offal at 0 (control), 20, 30, and 40% dietary inclusion levels (Table 1).

**Table 1:** Compositions of experimental diets.

**Ingredients (%)**	**Treatments**			
	**G1**	**G2**	**G3**	**G4**
Transgenic cottonseed meal	0	20	30	40
Wheat offal	40	20	10	0
Corn	28	28	28	28
Dehydrated lucerne meal	11	11	11	11
Soybean meal	16	16	16	16
Soybean oil	1.5	1.5	1.5	1.5
Calcium phosphate	2.3	2.3	2.3	2.3
Sodium chloride	0.7	0.7	0.7	0.7
Vitamins and mineral premix	0.5	0.5	0.5	0.5
Total	100	100	100	100

*G1 represent the control diet, G2, G3, and G4 represent 20, 30, and 40% inclusion of transgenic cotton seed respectively. Composition of premix Each Kg. Contains: Vitamin A, 15,000,000 IU; Vitamin B1, 5,000 mg; Vitamin C, 15,00 mg; Vitamin D3, 3,000,000 IU; Vitamin B2, 6,000 mg; Folic Acid, 750 mg; Vitamin E, 6,000 mg; Vitamin B6, 4,000 mg; Nicotinic Acid, 25,000 mg; Vitamin K3, 4,000 mg; Vitamin B12, 9,000 mcg; Calcium Pantothenate, 10,000 mcg*.

### Compositional analysis

Nutritional components of both control and experimental diets were analyzed according to the standard procedure explained by AOAC (24). Major compositions such as dry matter, moisture, crude protein, crude fiber, ether extract, neutral detergent fiber, acid detergent fiber, and hemicellulose were estimated. The total energy content of each diet was also measured (Table 2). All dietary ingredients were thoroughly mixed before subjecting to compositional analyses. Dry matter determination was achieved through drying the samples at 70°C for 24 h with the help of Memmert convection oven. Moisture was calculated using the formula: % Moisture = (100–%DM). The macro-Kjeldahl method was used for determination of the total nitrogen content. The determined nitrogen content was then converted to crude protein by multiplying with a factor of 6.25. Crude fiber was estimated as a fraction remaining following digestion with a standard solution of sulphuric acid (H_2_SO_4_) and sodium hydroxide (NaOH) in a controlled condition. Ash determination was achieved by ashing the samples at a high temperature of about 550°C in an oven for about 4 h. Soxhlet extraction method was used for ether extract determination. A formula: %NFE = %DM–(%CP + %CF + %EE + %Ash), was applied for the calculation of Nitrogen free extract. An automated fiber determination system (FIBRE TECH M System; Tecator, Sweden) was employed to determine both Neutral detergent fiber (NDF) and acid detergent fiber (ADF) as described by Van Soest et al. (25). Hemicellulose was calculated by applying formula; (%NDF–%ADF). An adiabatic bomb calorimeter was used for calculating the energy content.

**Table 2:** Compositional analyses of the experimental diets.

**Nutrients composition (%)**	**Treatments**			
	**G1**	**G2**	**G3**	**G4**
Dry matter	96.70	95.90	96.30	95.70
Moisture	3.93	4.14	3.73	4.34
Crude protein	15.70	15.90	16.50	17.60
Crude fiber	13.50	10.50	14.00	16.00
Ether extract	9.50	11.50	13.00	14.00
Ash	9.00	7.40	7.00	7.20
Nitrogen free extract	47.33	46.30	44.51	47.44
Neutral detergent fiber	60.31	59.75	61.22	60.17
Acid detergent fiber	37.05	37.59	38.13	38.32
Hemicellulose	23.28	22.18	23.15	21.93
Metabolizable energy(Kcal/g)	2,592	2,557	2,588	2,651

*All values in the table are average of the two independent measurements. G1 represent the control diet, G2, G3, and G4 represent 20, 30, and 40% inclusion of transgenic cottonseed respectively*.

### Experimental animals and their management

“A total of Forty-eight” New Zealand white male and female (24 pairs) rabbits weighing averagely 570.50 ± 0.50 g were purchased from Tollinton animals market Lahore, Pakistan. Just after receiving, all the animals were provided anti-stress (Symostress- **Product Code:** SSA100 gm) mixed with a clean drinking water for at least 3 days. The animals were allowed to acclimatize to the environment during the first 7 days before they were assigned to their respective treatments. All animals were kept at 22 ± 3°C (room temperature), under a period of 12 h (light/dark cycle) and (45–60%) humidity.

### Experimental procedure

Following seven days of the environmental adjustment, rabbits were balanced for weight and randomly allotted to four dietary treatment groups namely: G1, G2, G3, and G4 with 6 pairs per group (males and females is separate cages) for 6-month feeding trial. The rabbits in G1 were reared on a normal diet without GM cottonseed. Rabbits in G2, G3, and G4 were raised on diet containing 20, 30, and 40% of transgenic cottonseeds, respectively. One hundred and fifty grams (150 g) of the experimental diet was daily served to each animal to allowed for *ad-libitum* feeding. Clean water was made available throughout the experimental period.

### Feed consumption and body weight

**Figure 1:**
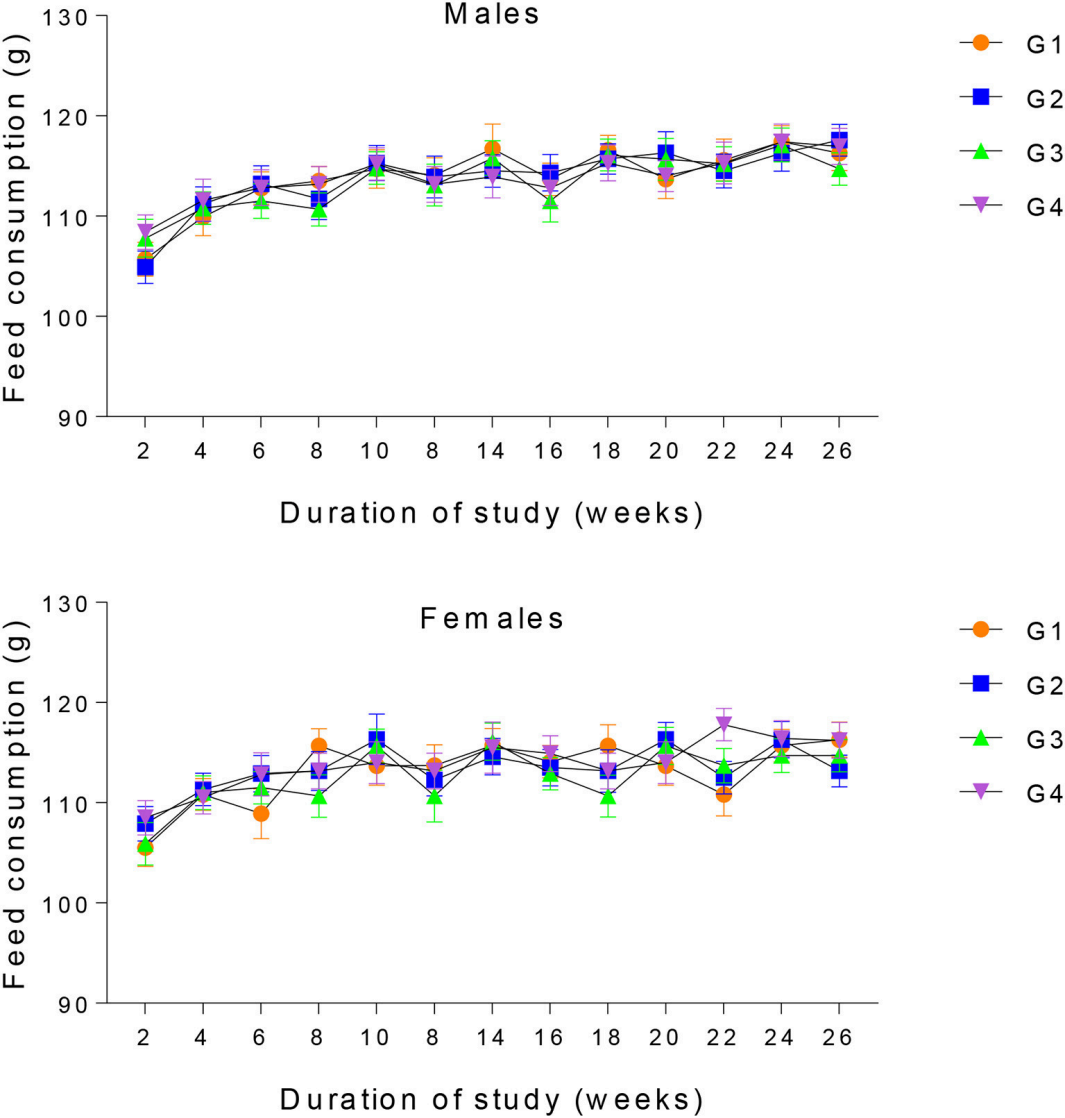
Mean feed consumption of male and female rabbits. G1 represent the Control group. G2, G3, and G4 represent 20, 30, and 40% GM cotton diet.

All animals were observed daily for any anomalies and mortality. The body weight of each animal was measured prior to dosing and this practice was routinely done on weekly basis throughout the study period. Feed intake for the animals in each group was determined daily by calculating the difference between the daily feed supplied and leftover. However, for the sake of understanding, the data on feed intake presented in Figure 1, were summarized on weekly basis.

Daily feed consumption (g) = (Amount of feed supplied−Amount of feed left).

### Blood collection and serum biochemistry

Blood samples were collected from the ear vein of each animal with a sterilized disposable syringe and needle. In order to minimize the standard error in values, the animals were fasted for 12 h (12 h) prior to blood collection. Three milli liter of blood sample was taken in a labeled sterile serum separator tubes at specific time intervals of 0, 45, 90, 135, and 180 days of the study. Serum was separated by centrifugation at 3,000 rpm at 4°C for 10 min and stored immediately at −20°C until use. The biochemical parameters which were measured include Serum enzymes, Serum electrolytes, Glucose, total cholesterol, Serum total protein, and Bilirubin. Among serum enzymes, aspartate aminotransferase (AST), alkaline phosphatase (ALP), alanine aminotransferase (ALT), creatinine, and urea (BUN) were studied. For serum electrolytes, levels of sodium [Na], potassium [K], calcium [Ca], and chloride [Cl] were measured. All the parameters were estimated in an automated biochemical analyzer (Accurex—Sphera Automated Clinical Chemistry Analyzer Italy), using commercial kits according to manufacturer instruction.

### Statistical analysis

Data on compositional analysis, feed consumption and weekly body weight were subjected to one-way analysis of variance (ANOVA). Observations of serum biochemical parameters were analyzed using two-way analysis of variance (2-way ANOVA). The diet, sampling period, and their interactions were considered. Data for male and female rabbits were analyzed separately, and results were presented as the mean ± standard error ($\bar{\text{x}}$ ± SEM). The mean of all the treatments was compared using “Tukey's Multiple Comparison Test” when appropriate for determining any significant differences. *P* < 0.05 were considered significant. All analysis was performed using (Graph Pad Prism software for Windows version 7).

### Bioethics

All experimental procedures were approved by the Ethics committee of National Centre of Excellence in Molecular Biology (CEMB), University of the Punjab) Lahore Pakistan.

## Results

### Compositional analysis

The proximate compositions of control and experimental deists are listed in Table 2. Although there are little variations of the calculated mean values in some of the proximate components, between control and the experimental diets, however, such differences are not statistically significant (*P* > 0.05).

### Body weight and feed consumption

**Figure 2:**
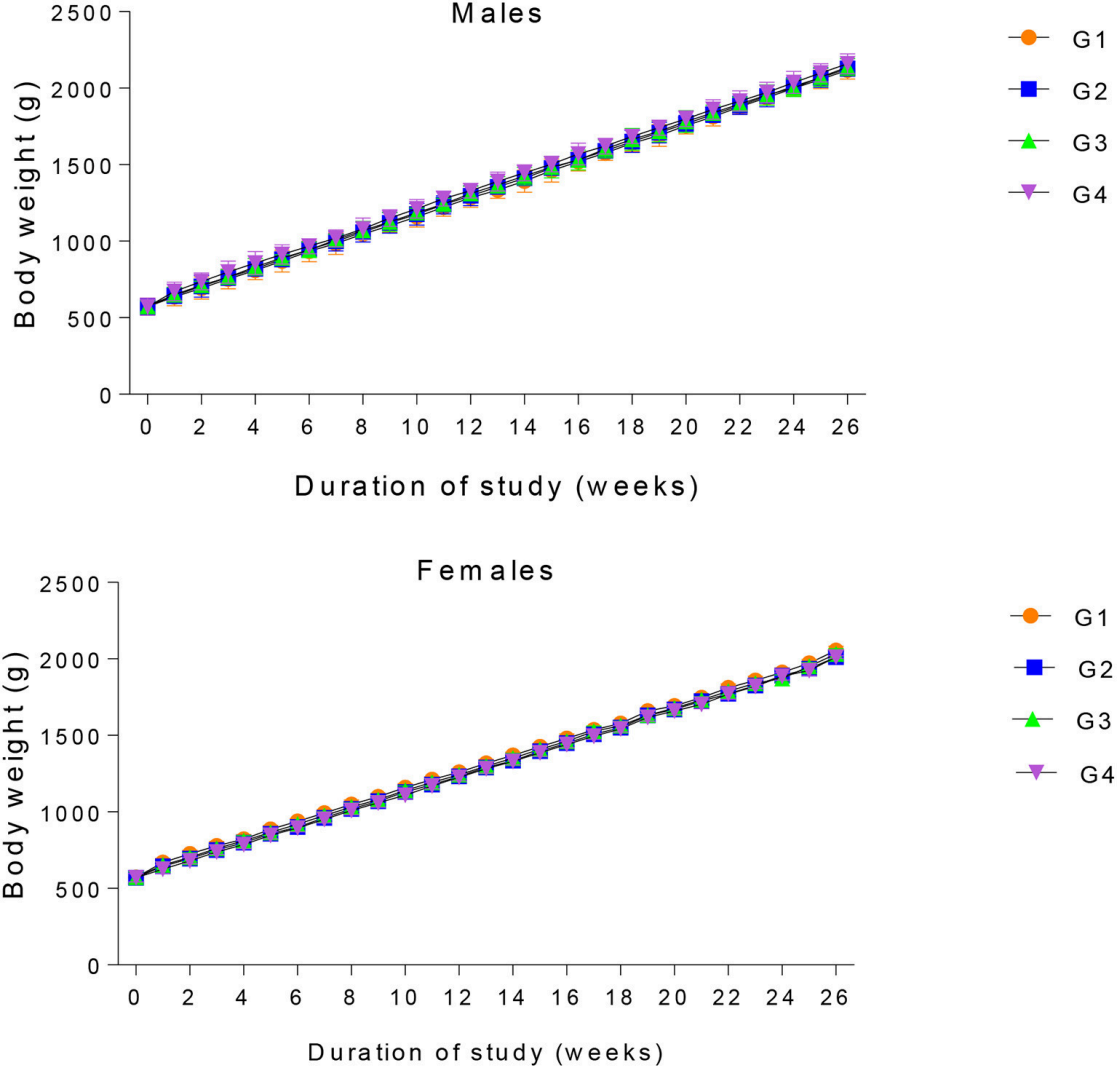
Mean weekly body weight of male and female rabbits throughout the growth phase. G1 represent the Control group. G2, G3, and G4 represent 20, 30, and 40% GM cotton diet.

With the passage of time, the body weight of each animal in all the four groups increases. No statistical differences were observed in the body weight of both control and experimental groups (Figure 2). Feed consumption was comparable among the four experimental groups throughout the course of the study (Figure 1).

### Serum biochemical changes and enzymes activities

The response and activities of serum enzymes such as aminotransferase (ALT), Aspartate transaminase (AST), alkaline phosphatase (ALP), Creatinine and Urea BUN in growing rabbits with respect to the dietary treatments are summarized in Tables 3, 4. The biochemical indices in all the four groups of the male and female rabbits after 180 days feeding trial were not significantly (*p* > 0.05) affected by dietary treatments. The level of urea BUN in the serum of male rabbit fed a diet containing 40% GM cotton seed was significantly higher (*P* < 0.05) at 135 days as compared with that of control and female rabbits. However, this increase was not observed in next 45 days. Apparent increase of urea BUN level in the serum of rabbits fed a diet containing 20 and 30% GM diet was also recorded but such increase was not statistically different from that of control groups.

**Table 3:** Effect of dietary *Bt* and *CP4EPSPS* proteins on Serum biochemical response and enzymes activities of male rabbits.

**Parameters**	**Days of sampling**	**Dietary treatments**				***P*****-values**		
		**G1**	**G2**	**G3**	**G4**	**Diet (T)**	**Period (P)**	T*P
ALT (U/l)	0	47.00 ± 9.42	49.17 ± 3.50	47.50 ± 6.64	47.67 ± 4.26	0.985	0.992	>0.999
	45	48.33 ± 5.06	51.83 ± 3.18	50.17 ± 3.38	48.17 ± 5.19			
	90	47.50 ± 4.94	49.33 ± 4.30	46.83 ± 5.78	49.00 ± 2.99			
	135	48.17 ± 7.44	47.50 ± 7.91	49.83 ± 6.73	94.17 ± 3.34			
	180	47.67 ± 5.11	47.17 ± 9.79	49.67 ± 4.96	47.83 ± 4.48			
AST (U/l)	0	96.00 ± 8.72	96.17 ± 6.67	95.83 ± 6.63	88.83 ± 7.84	0.693	0.914	0.999
	45	90.33 ± 5.67	91.50 ± 4.10	87.50 ± 9.87	91.33 ± 11.22			
	90	94.00 ± 5.01	97.83 ± 11.09	90.17 ± 11.24	85.17 ± 9.71			
	135	94.17 ± 6.42	90.83 ± 6.85	92.50 ± 7.46	84.17 ± 7.34			
	180	92.83 ± 5.27	95.00 ± 3.38	97.67 ± 4.72	89.67 ± 6.23			
ALP (U/l)	0	40.33 ± 3.50	41.00 ± 3.97	41.67 ± 4.66	40.00 ± 4.37	0.941	0.993	>0.999
	45	41.67 ± 4.93	41.83 ± 5.76	42.33 ± 10.12	42.83 ± 3.95			
	90	41.67 ± 3.78	42.50 ± 4.43	39.50 ± 2.64	43.33 ± 3.88			
	135	40.33 ± 5.71	40.67 ± 3.26	38.33 ± 6.26	43.83 ± 9.58			
	180	42.17 ± 7.87	37.83 ± 3.17	40.50 ± 5.76	42.50 ± 5.30			
UREA(mg/dl)	0	21.83 ± 1.72	22.00 ± 2.00	20.67 ± 1.48	21.00 ± 1.98	0.004	<0.0001	0.144
	45	20.33 ± 1.52	22.83 ± 1.89	23.17 ± 2.75	21.83 ± 1.44			
	90	21.67 ± 2.25	30.33 ± 2.59	32.00 ± 3.37	33.17 ± 3.51			
	135	22.67 ± 1.48	32.67 ± 4.45	33.50 ± 2.57	34.00 ± 2.93			
	180	21.83 ± 1.33	22.50 ± 1.23	21.50 ± 1.57	22.17 ± 1.58			
*CREA(mg/dl)	0	1.24 ± 0.13	1.22 ± 0.14	1.30 ± 0.19	1.36 ± 0.06	0.829	0.831	0.986
	45	1.33 ± 0.08	1.35 ± 0.10	1.30 ± 0.07	1.24 ± 0.12			
	90	1.32 ± 0.13	1.28 ± 0.09	1.43 ± 0.11	1.34 ± 0.13			
	135	1.35 ± 0.08	1.33 ± 0.08	1.31 ± 0.12	1.44 ± 0.08			
	180	1.34 ± 0.08	1.34 ± 0.08	1.43 ± 0.05	1.28 ± 0.14			

All values in the table are means ± SEM. G1 represent the Control group. G2, G3, and G4 represent 20%, 30%, & 40% GM cotton diet. *CREA; Creatinine. T^*^P; Interaction between diet and sampling periods.

**Table 4:** Effect of dietary *Bt* and *CP4EPSPS* proteins on Serum biochemical response and enzymes activities of female rabbits.

**Parameters**	**Days of sampling**	**Dietary treatments**				***P*****-values**		
		**G1**	**G2**	**G3**	**G4**	**Diet (T)**	**Period (P)**	T*P
ALT (U/l)	0	47.00 ± 6.39	48.17 ± 4.63	46.83 ± 5.11	47.17 ± 6.25	0.994	0.954	>0.999
	45	48.33 ± 3.95	50.67 ± 6.68	50.17 ± 8.10	48.00 ± 3.26			
	90	49.50 ± 5.14	48.17 ± 4.30	47.17 ± 5.31	49.50 ± 4.85			
	135	50.33 ± 3.37	47.50 ± 7.91	49.67 ± 7.94	51.50 ± 3.77			
	180	48.17 ± 5.53	46.83 ± 3.37	46.83 ± 4.60	48.50 ± 2.59			
AST (U/l)	0	91.33 ± 8.91	88.67 ± 11.99	95.33 ± 4.87	89.17 ± 7.06	0.98	0.997	>0.999
	45	87.00 ± 10.27	92.17 ± 6.85	87.50 ± 9.87	92.33 ± 13.44			
	90	91.17 ± 5.42	93.17 ± 12.19	90.17 ± 11.24	86.33 ± 11.70			
	135	89.17 ± 7.17	90.00 ± 6.28	92.67 ± 5.67	86.67 ± 5.17			
	180	92.83 ± 11.59	90.33 ± 8.51	90.83 ± 3.15	91.50 ± 3.52			
ALP (U/l)	0	41.83 ± 5.27	42.00 ± 3.34	41.33 ± 3.49	40.50 ± 3.56	0.938	0.996	>0.999
	45	40.50 ± 4.07	41.67 ± 3.68	42.00 ± 5.12	40.17 ± 4.90			
	90	41.33 ± 3.68	43.33 ± 2.06	41.00 ± 3.06	41.17 ± 3.85			
	135	39.83 ± 3.24	41.83 ± 7.27	42.67 ± 5.63	38.83 ± 5.71			
	180	41.83 ± 5.28	39.17 ± 5.76	41.33 ± 5.94	39.50 ± 2.94			
UREA(mg/dl)	0	20.00 ± 2.56	20.50 ± 1.65	19.17 ± 2.68	18.00 ± 2.03*a*	0.024	<0.0001	0.257
	45	21.67 ± 1.54	23.33 ± 2.17	22.67 ± 2.78	23.33 ± 1.98			
	90	24.83 ± 2.93	32.667 ± 3.27	32.50 ± 4.18	29.33 ± 3.19			
	135	20.50 ± 1.36	33.83 ± 5.95	30.67 ± 2.88	32.50 ± 3.66			
	180	22.67 ± 1.78	21.83 ± 1.66	30.17 ± 1.99	24.33 ± 2.04			
*CREA(mg/dl)	0	1.23 ± 0.14	1.45 ± 0.11	1.44 ± 0.13	1.30 ± 0.13	0.757	0.996	0.986
	45	1.33 ± 0.08	1.34 ± 0.09	1.30 ± 0.11	1.37 ± 0.09			
	90	1.32 ± 0.06	1.38 ± 0.10	1.35 ± 0.10	1.39 ± 0.09			
	135	1.37 ± 0.11	1.40 ± 0.10	1.30 ± 0.18	1.31 ± 0.10			
	180	1.26 ± 0.14	1.31 ± 0.10	1.42 ± 0.03	1.38 ± 0.10			

All values in the table are means ± SEM. G1 represent the Control group. G2, G3, and G4 represent 20%, 30%, & 40% GM cotton diet. *CREA; Creatinine. T^*^P; Interaction between diet and sampling periods.

### Serum electrolytes

The response of various serum electrolyte like Na, K, Ca, and Cl of male and female rabbits were studied following feeding with four different types of diets for 180 days. Tables 5, 6 summarized the analyzed values in all the groups as mean ±SEM. The basal level of serum Na was statistically the same in all the groups and remained unchanged during the course of the study. Similarly, the initial level of serum K and Ca were comparable in the four of the experimental groups. The response of serum Cl to dietary treatments was also evaluated and all the values remained unaltered in the control as well as in the treated groups of rabbits.

**Table 5:** Effect of dietary *Bt* and *CP4EPSPS* proteins on the Serum electrolyte of male rabbits.

**Parameters**	**Days of sampling**	**Dietary treatments**				***P*****-values**		
		**G1**	**G2**	**G3**	**G4**	**Diet (T)**	**Period (P)**	T*P
Na(mEq/l)	0	139.00 ± 6.23	138.50 ± 7.24	139.17 ± 5.73	138..33 ± 4.11	0.945	0.816	>0.999
	45	139.67 ± 3.25	140.00 ± 5.37	139.00 ± 3.39	141.17 ± 3.33			
	90	142.50 ± 2.77	142.17 ± 2.55	139.67 ± 4.54	142.17 ± 2.99			
	135	139.50 ± 2.93	142.67 ± 4.48	143.00 ± 4.74	142.83 ± 3.54			
	180	139.50 ± 3.79	142.00 ± 2.81	13983 ± 3.60	144.00 ± 2.70			
K (mEq/l)	0	4.68 ± 0.47	5.18 ± 0.55	5.05 ± 0.59	5.22 ± 0.60	0.995	0.447	0.999
	45	4.50 ± 0.43	4.58 ± 0.59	4.53 ± 0.86	4.30 ± 0.47			
	90	4.40 ± 0.42	4.43 ± 0.32	4.52 ± 0.40	4.38 ± 0.47			
	135	4.85 ± 0.77	4.48 ± 0.55	4.60 ± 0.45	4.63 ± 0.57			
	180	5.03 ± 0.44	4.62 ± 0.28	4.67 ± 0.31	5.20 ± 0.71			
Ca(mEq/l)	0	10.05 ± 0.62	9.95 ± 0.67	10.03 ± 1.24	9.83 ± 0.92	0.977	0.986	>0.999
	45	10.15 ± 0.67	9.58 ± 0.96	9.48 ± 1.65	10.20 ± 0.67			
	90	10.12 ± 0.68	10.02 ± 0.69	9.68 ± 1.06	10.00 ± 0.95			
	135	9.80 ± 0.29	9.82 ± 0.93	9.80 ± 1.07	10.03 ± 0.67			
	180	9.61 ± 0.66	9.73 ± 0.87	9.63 ± 0.85	9.58 ± 0.64			
Cl (mEq/l)	0	101.17 ± 3.66	99.83 ± 3.15	100.33 ± 4.30	101.50 ± 4.06	0.537	0.794	0.999
	45	120.83 ± 3.34	101.50 ± 2.63	102.17 ± 4.02	103.67 ± 3.37			
	90	101.83 ± 3.32	101.83 ± 4.56	102.33 ± 3.42	104.33 ± 5.30			
	135	103.50 ± 5.74	103.00 ± 3.59	101.17 ± 4.78	109.17 ± 5.12			
	180	102.50 ± 4.19	105.50 ± 4.60	100.33 ± 2.06	106.17 ± 5.17			

All values in the table are means ± SEM. G1 represent the Control group. G2, G3, and G4 represent 20, 30, and 40% GM cotton diet. Na; Sodium, K; Potassium, Ca; Calcium, Cl; Chloride. T^*^P; Interaction between diet and sampling periods.

**Table 6:** Effect of dietary *Bt* and *CP4EPSPS* proteins on the Serum electrolyte of female rabbits.

**Parameters**	**Days of sampling**	**Dietary treatments**				***P*****-values**		
		**G1**	**G2**	**G3**	**G4**	**Diet (T)**	**Period (P)**	T*P
Na (mEq/l)	0	140.50 ± 4.02	140.33 ± 6.08	141.17 ± 4.95	140.67 ± 2.99	0.971	0.837	>0.999
	45	140.00 ± 3.67	139.50 ± 3.39	138.17 ± 2.70	141.67 ± 3.02			
	90	141.33 ± 4.99	143.17 ± 5.93	141.67 ± 4.18	143.33 ± 4.01			
	135	142.17 ± 3.67	143.33 ± 2.82	142.83 ± 4.28	143.00 ± 2.67			
	180	142.17 ± 3.29	141.83 ± 4.74	140.33 ± 3.70	141.50 ± 4.22			
K (mEq/l)	0	5.23 ± 0.59	5.32 ± 0.63	5.18 ± 0.51	4.90 ± 0.55	0.874	0.519	0.998
	45	4.27 ± 0.29	4.78 ± 0.64	4.72 ± 0.70	4.53 ± 0.42			
	90	4.50 ± 0.39	4.65 ± 0.56	4.65 ± 0.74	4.20 ± 0.39			
	135	4.87 ± 0.66	5.35 ± 0.71	4.32 ± 0.30	5.27 ± 0.68			
	180	4.80 ± 0.60	4.83 ± 0.58	4.92 ± 0.61	4.58 ± 0.50			
Ca (mEq/l)	0	9.83 ± 0.51	9.92 ± 0.75	10.00 ± 0.71	9.93 ± 0.67	0.995	0.988	>0.999
	45	10.23 ± 0.81	9.47 ± 0.54	9.37 ± 0.70	10.05 ± 0.84			
	90	9.50 ± 0.83	9.92 ± 1.02	9.70 ± 0.41	9.70 ± 0.88			
	135	9.90 ± 1.15	9.50 ± 1.02	9.58 ± 0.52	9.52 ± 1.31			
	180	9.60 ± 0.89	9.67 ± 0.57	9.87 ± 0.36	9.58 ± 0.91			
Cl (mEq/l)	0	100.17 ± 3.36	100.50 ± 3.29	101.50 ± 4.65	102.67 ± 5.16	0.977	0.938	>0.999
	45	105.33 ± 4.34	104.17 ± 3.07	102.67 ± 4.20	101.50 ± 4.61			
	90	103.17 ± 5.88	103.83 ± 5.12	102.17 ± 5.24	10.00 ± 3.24			
	135	103.33 ± 4.78	104.50 ± 3.64	101.00 ± 2.32	101.33 ± 3.67			
	180	102.50 ± 4.21	104.17 ± 4.30	104.33 ± 4.54	102.83 ± 4.21			

All values in the table are means ± SEM. G1 represent the Control group. G2, G3, and G4 represent 20, 30, and 40% GM cotton diet. Na; Sodium, K; Potassium, Ca; Calcium, Cl; Chloride. T^*^P; Interaction between diet and sampling periods.

### Serum total cholesterol and glucose

**Figure 3:**
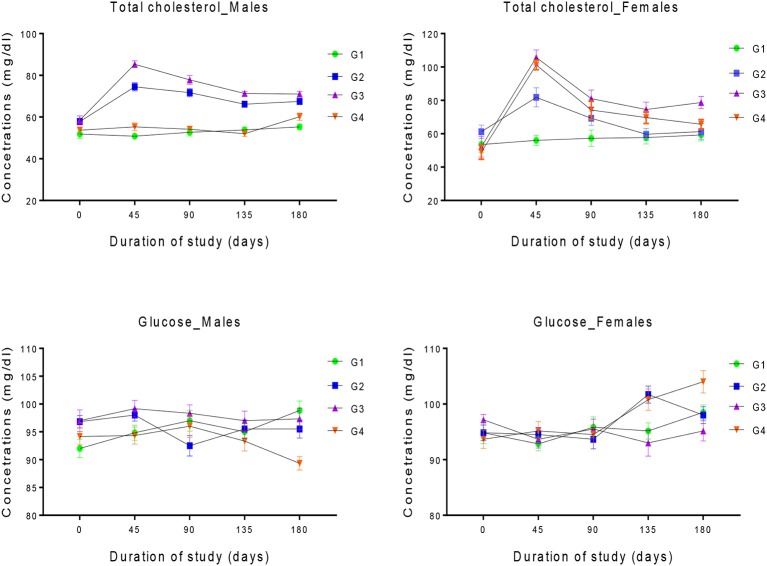
Changes in the levels of serum total cholesterol and Glucose in the four experimental groups of male and female rabbits. group. G1 control group, G2, G3, and G4 represent 20, 30, and 40% GM cottonseed respectively. Two-way ANOVA indicated significant differences between groups, sampling periods and their interaction (*P* < 0.05). *Post-hoc* multiple comparisons further revealed significant differences between specific treatment groups and the control groups.

Figure 3 represent the effect of feeding GM cottonseed on the serum total cholesterol and glucose of each rabbit after 180-day supplement. The initial levels of serum total cholesterol in all the four groups of male rabbits were not statistically different (*p* > 0.05) from one other. However, following 45 days of GM cottonseed consumption, significant increased (*P* < 0.01) were observed in the levels of serum total cholesterol of G3 as compared with other groups.

The level of the serum total cholesterol of G2 was also found to be significantly higher (*P* < 0.01) than those in G1 and G2 at 45 days of the study. The serum levels of total cholesterol in both G1 and G4 remained significantly lower when compared to those of G2 and G3 from 45 days until the end of the study. The basal level of serum total cholesterols in G3 female rabbits was significantly higher (*P* < 0.05) than those in the remaining groups. A significant increase in serum total cholesterol was recorded in all the groups of female rabbits with exception of the control groups at 45 and 90 days of the study. The cholesterol level in G3 and G4 were also significantly higher as compared with G2 at 45 days. The values of serum total cholesterol of G1 and G2 female rabbits were similar at 135 and 180 days but significantly lower than those in G3 and G4. The cholesterol levels in G4 were also significantly less when compared with G3 at 180 days. Similarly, no significant differences were found (*P* < 0.05) in the initial levels of serum glucose in all the groups of male and female rabbits. The levels continued to be the same in male rabbit of all the groups until after 180 days in which the level in G4 was significantly lower as compared with G1 and G3. Similar trend seems to be observed in the female rabbits for all the groups. The glucose levels of G3 female rabbit at 135 days were significantly lower as compared with those of G2 and G4. After 135 days the levels in all the groups were statistically equal except in G4 which is significantly higher (*P* < 0.05) than that in G3.

### Serum proteins

**Figure 4:**
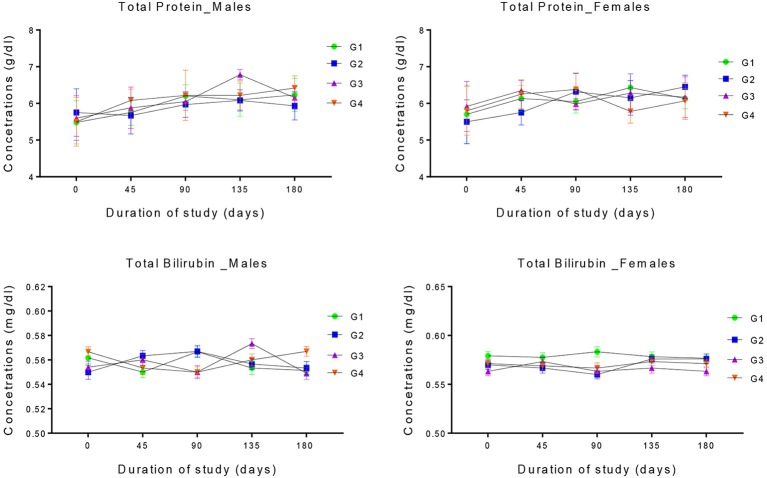
Changes in the levels of serum total Protein and total Bilirubin in the four experimental groups of male and female rabbits. group. G1 control group, G2, G3, and G4 represent 20, 30, and 40% GM cottonseed respectively. Two-way ANOVA indicated non-significant differences between groups, sampling periods and their interaction (*P* >0.05).

The response of serum total protein and total bilirubin of growing male and female rabbits fed different levels of dietary GM cottonseed meal is presented in the Figure 4. There were no significant differences (*P* > 0.05) in the basal levels of serum total proteins of all the groups. These levels remained consistently the same throughout the studies. The initial and final values of serum total bilirubin of all the groups were also equal. Even though there were some differences in the calculated mean values yet these differences were not statistically significant (*P* > 0.05).

## Discussion

Genetically modified (GM) crops have huge potential in plant production, however, their safety as food and feed remains debatable. Various Conflicting reports and controverting opinions were raised continuously regarding the potential risks of GM crops on both human and animal health. Nevertheless, very little is known about the validity of the various safety assessments tests that were performed for these crops (3). Intensive testing of transgenic crops in animal models together with performance and toxicology assessment for substantial equivalence was still suggested (26, 27). Though cotton seeds are not directly consumed by human being rather their byproducts but still farm animals ingest considerable quantities of plant material and there are significant chances for the livestock to consume substantial amounts of transgenic DNA and protein which may lead to alteration in their hematological and biochemical profile (28). Thus, this study was conducted to assess the effect of feeding transgenic cottonseed expressing *Bt* and *Cp4epsps* proteins on serum biochemical profile of rabbits.

The principle of substantial equivalence has recently been considered as a means of food safety assessment according to the international standard of transgenic crops (29–32). On basis of this principle, assessment of nutritional components between the GM crop and their non-GM counterpart is an essential factor for safety evaluation of GM food/feed product (33). In the present study, the dietary intake of transgenic cottonseed was evaluated via the nutritional response on rabbits. The major nutritional components of control and experimental diets were analyzed before feeding to the rabbits. No statistical differences were observed in nutrient compositions between control and experimental diets. The result of this study indicated that incorporation of transgenic cotton seed of up to 40% does not result in significant changes of chemical compositions of the diets. Tripathi and his coworkers reported that integration of *Bt-genes* expressing insect resistant proteins (*Cry1Ac, Cry2Ab, Cry2Ab2*) or *Cp4 epsps* protein could not modify the cottonseed‘s chemical compositions (28). Studies have also shown that the chemical compositions of cotton plants that were genetically modified to express different types of genes like *Cry1Ac, Cry2Ab*, and *Cp4 epsps* gene or combinations of *Cry1Ac* and *Cry2Ab* genes; *Cry1Ac* and *Cp4 epsps* genes, were found to be identical (34).

The feed intake and mean weekly body weights of animals in all the four groups were not affected by the dietary treatments. The result of this study signified that feeding transgenic cotton seed even at 40% level of inclusion did not have detrimental influence on growth and development of the animals. The result of this study is in accordance with the previous findings indicating non-significant differences in body weight of rabbits when 30% of *Bt* cotton seeds were incorporated in their diets (17). Our finding thus, agreed with the recommendations of previous researchers that addition of GM cotton in animal diet is safe since the presence of the transgenes in the cotton genome does not account for significant changes in its nutritional components (26, 27, 35–37). Similar results were also observed, when catfish and Northern Bobwhite Quail were exposed to *Bt* cottonseed meal (38–40). Moreover, researches have conveyed that feed consumption and body weight of lactating Holstein cows were not significantly altered when they were fed a ration containing whole GM cottonseed (34).

In serum enzymology, enzymes concentration is used to diagnose the impairment in heart, liver, and kidney, providing a valuable information with respects to their state of damage (41). Serum enzymes such as aminotransferases are intracellular enzymes which are normally found at low level in plasma and control the release of cellular content during the normal cell renewal (42). It has been reported in numerous studies that higher level of ALT and AST is mostly caused by Liver infections resulting in extensive cell death, as well as severe viral hepatitis or toxic liver damage (42). In this study, the mean values of all the tested serum enzymes of both male and female rabbits were evaluated and results indicated that there were no significant differences in LFTs of each group and all the values were within the normal reference range reported for rabbits (43). The results of serum enzyme activities obtained in this study clearly determined that, exposing the rabbits to dietary GM cottonseeds for 180 days have no adverse effect in the function of their vital organs like liver, kidneys and heart. These results are in line with the report of Yonemochi et al. (44), who found no detrimental effect of GM maize on serum lactate dehydrogenase levels in dairy cows. Similar results were also reported by Tudisco et al. (45), that GM soya bean did not affect the several specific enzymes in serum, heart, skeletal muscle, liver, and kidney of the experimental animals. On the contrary, Singh and his coworkers reported the decreased levels of ALT in serum of buffaloes raised on GM cottonseed (46). However, the authors concluded that there was found no health hazardous effect on buffaloes as confirmed from the hematological indices.

The serum electrolytes such as Na, K, Ca, and Cl are the minor dietary nutrients, which are reported to be least significantly influenced by changes in diet compositions. Nevertheless, their great clinical significance cannot be overlook (47). Moreover, they played a significant role in most of the daily body functions like nerve conduction, heart and skeletal muscle contraction, acid-base balance, osmotic pressure regulation, monosaccharide and amino acid absorption (48–50). The results of serum electrolytes reported in this study are in accordance with reports and all values fall within the standard reference range as reported by Jones (43). The results obtained from this study revealed that the electrolytes balance was not influenced due to consumption of GM diet. Similar conclusions have been reported by Zhou et al. (51), when Sprague–Dawley rats were fed high-amylose transgenic rice for up to three generations. The findings of this study corroborate with that of Prasad (52) who fed rabbits with a high-cholesterol diet for 6-months. However, on the contrary (42), reported significant changes in the levels of serum electrolytes of rabbits following 6-weeks consumption of dietary fumonisin.

The cholesterol is the principal type of sterol biosynthesized by all animal cells, as an essential structural component of all animal cell membranes, and precursor of steroid hormones, vitamin D and bile acids (53). The cholesterol is not just a simple waste product of metabolism but it functions as an important cell protective barriers for dehydration and infection (54). Similarly, glucose is the major source of energy not only for erythrocytes but also act as an essential short-term energy source for many others tissues e.g., central nervous system (55). Although, significant differences between the groups were calculated in the level of serum total cholesterol and glucose in some of the experimental male and female rabbits following 180-day feeding trial with GM cottonseeds. However, these differences were not biologically significant since all the disparities were within the reported normal physiological reference values of rabbits (43). The result of this experiment is therefore suggesting that the differences observed could not be attributed due to exposing the animal to GM cottonseed but could be as a result of some other physiological factors which are specific to individual animals. This result, validate the finding of (17) who found non-significant differences in the levels of serum total cholesterol and glucose when rabbits were fed with GM cotton seeds and fresh leaves for 90 days. Similarly, (33) reported non-significant differences in the levels of serum total cholesterol and glucose when glyphosate-resistant transgenic soybean GTS40-3-2 was fed to rats for 195 days. Also, non-significant differences were reported in the levels of triglyceride and total cholesterol when GM and conventional rice was fed to rats for a 90-day study (56).

Studies have shown that different types of the dietary proteins such as total proteins, albumin, globulin and total bilirubin are affected generally by total protein intake (57, 58). The initial and final values of all the protein types studied in this experiments were not statistically different. The non-significant differences recorded for the serum total proteins and bilirubin in this study, is an indication that the dietary *epsps and Bt* proteins have not adversely affect the protein metabolism in the experimental animals, since the synthesis of serum protein is mainly associated with the quantity of the available protein in the diet (59). The nutritional adequacy of the dietary proteins was also revealed based on the results obtained. The findings of this study were in agreement with the report of Rahman et al. and Yuan et al. (17, 33).

## Conclusion

No adverse effects were observed on the serum biochemical of rabbits following feeding with a diet containing different levels (20, 30, and 40%) of genetically modified cottonseed harboring *Bt* and *EPSPS* genes for 180 days. The findings of this study, thus suggested that the GM cottonseed meal could be used safely as a feed ingredient like other conventional feed products.

## Author contributions

The concept and design of the experiment was initiated by IS. IS, AS, and TH: Preparation of equipment and materials; IS and AR: Review of literature; IS and QA: Data collection and analysis; IS, AS, and QA: Preparation of main manuscript; QA and AR: Critical review of manuscript; IS, AS, QA, AR, and TH: Approval of the final version of manuscript. The manuscript was read by all authors and approved for publication.

### Conflict of interest statement

The authors declare that the research was conducted in the absence of any commercial or financial relationships that could be construed as a potential conflict of interest.
